# AMPK potentiation by LKB1 isoforms

**DOI:** 10.18632/oncotarget.6127

**Published:** 2015-10-15

**Authors:** Chantal Thibert, Christine Perret, Marc Billaud

**Affiliations:** Institut Albert Bonniot, CRI INSERM/UJF U823 Université Grenoble Alpes, Grenoble, France; INSERM U1016, Institut Cochin, Paris, France

**Keywords:** LKB1, ΔN-LKB1, isoforms, AMPK, metabolism

Cells must finely tune their metabolic pathways to face variations of nutrient fluxes. This dynamic maintenance of homeostasis is a fundamental requisite constraining all cellular processes. The serine/threonine kinase LKB1 is a hub of a molecular network monitoring the cellular energy charge via the phosphorylation and activation of the AMP-activated kinase (AMPK) [1]. In conditions of metabolic stress that increases the AMP/ATP ratio, AMPK is activated and switches off anabolic pathways (protein, cholesterol and lipid synthesis) while switching on catabolic pathways (glycolysis and fatty acid oxidation) to replenish the intracellular pool of ATP [1]. The central role of the LKB1-AMPK pathway is attested by the fact that *LKB1* is a tumor suppressor gene whose mutational inactivation is associated with a cancer-inherited condition and several sporadic malignancies [1].

The *LKB1* locus encodes two isoforms, the ubiquitous full-length form of the protein (LKB1L) [1] and a splice variant that differs in its C-terminal part (LKB1S) and is mostly expressed in male haploid germ cells [2]. Each of these two proteins binds separately to the pseudokinase STRAD and to the scaffolding molecule MO25 to form a heterotrimer that allosterically stimulates LKB1 enzymatic activity [1]. One lingering question is whether AMPK is constitutively phosphorylated by LKB1 or whether this process is regulated in response to microenvironmental cues. Several posttranslational modifications of LKB1 are proposed to affect its ability to activate AMPK [1, 3-6], but other mechanisms may be at play and this topic is the current subject of investigation.

We recently reported the identification of a third isoform of LKB1 produced upon alternative splicing in exon 1 that eliminates the start codon and favors the use of an in-frame translation initiation site situated downstream in exon 3 [7]. The resulting isoform, called ΔN-LKB1, is a truncated protein that is deleted of the nuclear localization signal and the N-terminal lobe of the catalytic domain. Accordingly, the location of ΔN-LKB1 is restricted to the cytoplasm and this isoform is devoid of enzymatic activity. Furthermore, ΔN-LKB1 is neither able to bind to STRAD nor to MO25. ΔN-LKB1 is mostly expressed in heart and skeletal muscles, and since these tissues are characterized by their high oxidative capacity, this observation prompted us to explore the putative metabolic functions of ΔN-LKB1. Surprisingly, ΔN-LKB1 potentiates LKB1-induced activation of AMPK and this effect is dependent of the catalytic activity of LKB1. Consistently, expression of ΔN-LKB1 in the liver of fasted mice enhances AMPK phosphorylation, stimulates fatty acids oxidation and inhibits triglyceride synthesis. Hence the question is how ΔN-LKB1 stimulates AMPK functioning. We established that ΔN-LKB1 binds with a higher affinity than LKB1 to the catalytic subunit of AMPK (the holoenzyme is made of three chains, the α catalytic subunit and two regulatory subunits, β and γ). Furthermore, ΔN-LKB1 interacts specifically with the autoinhibitory domain (AID) of AMPK whereas the binding of LKB1 to AMPK is not significantly impaired by the removal of the AID. This motif is a short amino acid stretch of the α chain polypeptide that interferes with the fixation of substrates in the catalytic pocket and whose inhibitory function is relieved upon binding of AMP to the AMPK γ subunit. Thus, ΔN-LKB1 may induce a conformational change of AMPK that destabilizes the AID and favors LKB1-dependent phosphorylation of AMPK that eventually triggers its activation.

**Figure 1:**
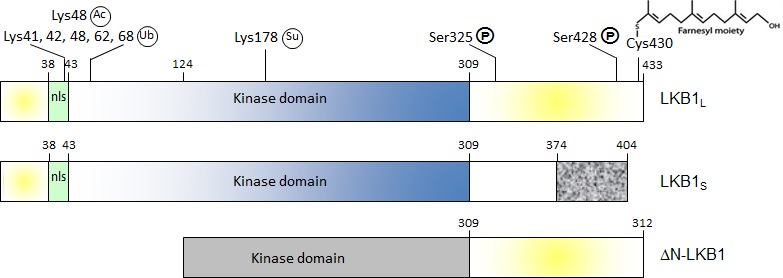
Schematic representation of human LKB1 isoforms

While further exploring ΔN-LKB1 properties, we found that this isoform is endowed with an intrinsic transforming ability as revealed by classical clonogenic and anchorage-independent cell growth assays [7]. Moreover, the silencing of Δ*N-LKB1* in a lung cancer cell line carrying a homozygous mutation of *LKB1* that disrupts expression of LKB1L but preserves expression of ΔN-LKB1 markedly reduces the viability of these tumor cells and impairs their growth when grafted to nude mice [7].

What might be the physiological functions of ΔN-LKB1? Interestingly, AMPK is known to maintain the level of NADPH through the phosphorylation and inhibition of acetyl-CoA carboxylases (ACC1 and ACC2), two major rate-controlling enzymes of the fatty acid biosynthetic pathway [8]. NADPH has a key role in neutralizing reactive oxygen species (ROS) and the ensuing deleterious oxidative stress. Since high levels of ROS are produced during intense muscle exercise, ΔN-LKB1 may limit the increase of ROS in this tissue by reducing ACCs activity via the enhancement of AMPK activity. Furthermore, AMPK is known to support the survival of malignant cells and to inhibit anoïkis, an apoptotic process induced by the detachment of cells from the extracellular matrix, via the increase of the NADPH/NADP ratio [8]. Therefore, ectopic expression of ΔN-LKB1 in tumor cells together with LKB1 or another AMPK kinase when LKB1 is mutationally inactivated may preserve the redox balance through AMPK activation, thereby fostering cancer development in certain contexts. Accordingly, AMPK can both suppress and promote neoplastic outgrowth depending on the stage of the tumor [1]. Collectively, these findings sustain the idea that the activation of AMPK by LKB1 can be modulated through the expression of ΔN-LKB1, and further provide unexpected evidence that LKB1 codes for isoforms with seemingly antagonistic roles, either oncosuppressive or pro-oncogenic.
